# Automatic Segmentation of Teeth, Crown–Bridge Restorations, Dental Implants, Restorative Fillings, Dental Caries, Residual Roots, and Root Canal Fillings on Orthopantomographs: Convenience and Pitfalls

**DOI:** 10.3390/diagnostics13081487

**Published:** 2023-04-20

**Authors:** Emel Gardiyanoğlu, Gürkan Ünsal, Nurullah Akkaya, Seçil Aksoy, Kaan Orhan

**Affiliations:** 1 Department of Dentomaxillofacial Radiology, Faculty of Dentistry, Near East University, 99138 Nicosia, Cyprus; 2 DESAM Institute, Near East University, 99138 Nicosia, Cyprus; 3 Department of Computer Engineering, Applied Artificial Intelligence Research Centre, Near East University, 99138 Nicosia, Cyprus; 4 Department of Dentomaxillofacial Radiology, Faculty of Dentistry, Ankara University, 06560 Ankara, Turkey

**Keywords:** automatic segmentation, orthopantomography, artificial intelligence, deep learning

## Abstract

Background: The aim of our study is to provide successful automatic segmentation of various objects on orthopantomographs (OPGs). Methods: 8138 OPGs obtained from the archives of the Department of Dentomaxillofacial Radiology were included. OPGs were converted into PNGs and transferred to the segmentation tool’s database. All teeth, crown–bridge restorations, dental implants, composite–amalgam fillings, dental caries, residual roots, and root canal fillings were manually segmented by two experts with the manual drawing semantic segmentation technique. Results: The intra-class correlation coefficient (ICC) for both inter- and intra-observers for manual segmentation was excellent (ICC > 0.75). The intra-observer ICC was found to be 0.994, while the inter-observer reliability was 0.989. No significant difference was detected amongst observers (*p* = 0.947). The calculated DSC and accuracy values across all OPGs were 0.85 and 0.95 for the tooth segmentation, 0.88 and 0.99 for dental caries, 0.87 and 0.99 for dental restorations, 0.93 and 0.99 for crown–bridge restorations, 0.94 and 0.99 for dental implants, 0.78 and 0.99 for root canal fillings, and 0.78 and 0.99 for residual roots, respectively. Conclusions: Thanks to faster and automated diagnoses on 2D as well as 3D dental images, dentists will have higher diagnosis rates in a shorter time even without excluding cases.

## 1. Introduction

Technological changes have created great changes in the fields of medicine and dentistry, and one of the most important innovations that caused this change is artificial intelligence (AI) technology. AI will come to be increasingly preferred in the fields of medicine and dentistry due to its important contributions to patient health services and the convenience that it provides to practicians. The increase in processing speed, computing power, storage capacity, ability to perform different tasks, and the affordability of advanced graphics processing units as well as computers are considered the beginning of a new era in medicine and especially in radiology [1,2,3].

Artificial intelligence (AI) is most simply defined as systems that mimic human intelligence to perform specific tasks and can improve themselves by repeating the data that they process. AI systems can display behaviors associated with human intelligence, such as planning, learning, reasoning, problem solving, perception, movement, manipulation, and, to a lesser extent, social intelligence and creativity [4,5,6,7,8]. Machine learning is considered to be a type of AI that can reveal results even for that which it was not programmed for. In 1959, Arthur Samuel defined machine learning as “the ability of machines to learn results for which they were not specifically programmed” [9]. He also developed a checkers game that can work in a computer environment, learn from its own mistakes, and thus improve itself [1,10,11,12].

One of the applications that made machine learning popular is image recognition. For image recognition, in order for a machine to learn what a relevant image is, it needs to be trained by showing similar images as much as possible [13,14,15,16]. Thus, by recognizing similar sequences, similar motifs, and similar pixels, the machine can detect, segment, and classify what those pictures are. The more data there are, the better artificial intelligence features will be revealed [1,11,12,17,18,19,20,21,22,23,24,25,26,27,28,29]. Deep neural networks have many hidden layers, with millions of interconnected artificial neurons. A number, called weight, represents the connections between one node and another [11,12,30,31,32]. Theoretically, deep neural networks can match any type of input to any type of output; however, they require much more training compared to other machine learning methods. They require millions of samples, compared to the hundreds or thousands of training data samples that a simpler network might need [1,2,11,12,17,18,19,20,21,30,33,34,35,36,37,38,39].

AI has quickly become more well-known and significant in dentistry clinical research. AI applications that can alter the conventional organization of dentistry education in universities remain in development [40,41,42]. Many technology advancements are now being quickly incorporated into the difficult dental training phase, from AI-supported virtual reality simulations to robotic patients [43,44]. Clinical decision support systems are a valuable aspect of computer technology that lower clinical inaccuracies, assist doctors in making better and more effective choices regarding patient care, and ultimately improve the standard of care [6,45]. This innovation has the potential to be an effective tool for clinical teaching in dental schools since it may provide medical professionals and students with more confidence in the course of treatment [46,47,48,49]. AI can be used to diagnose many diseases in many countries with poor socioeconomic statuses. For example, in countries with a high prevalence of tuberculosis, it is sometimes impossible for patients to be diagnosed due to the small number of radiologists who will evaluate the images of patients. Because artificial intelligence can accurately diagnose pulmonary tuberculosis with 95% sensitivity and 100% specificity, it can be easily diagnosed even in countries where radiologists are available with artificial intelligence by evaluating radiographs to be loaded from health centers [15,32,50,51,52].

Common applications of AI in oral diagnosis and dentomaxillofacial radiology are as follows:

- Oral cancer prognosis and assessment of oral cancer risk [45,53,54,55,56,57,58,59,60,61,62];
- Determination of temporomandibular joint disorder progression and temporomandibular internal derangements [27,30,34,38,63];
- Interpretation of conventional 2D imaging [31,64,65,66,67,68];
- Interpretation of cone beam computed tomography and other 3D imaging methods [1,10,12,17,18,19,21,23,27,69,70,71].

The majority of the research was designed to address dental issues; periapical radiography, orthopantomography (OPG), and lateral cephalometric radiographs represent the most frequent imaging techniques in dentomaxillofacial radiology. One of the earliest experiments using a 3D imaging AI model sought to distinguish between radicular cysts and apical granulomas [72]. Cephalometric landmark detection, osteoporosis analysis, odontogenic lesion categorization, and the detection of periapical/periodontal pathologies are the most frequently studied topics for AI in DMFR, according to a review by Hung et al. [7].

The majority of the studies in DMFR are about the “localization” and “basic features of teeth”, rather than general evaluations. Although the focus is on three-dimensional radiographs, such as cone beam computed tomography (CBCT), and two-dimensional radiographs, such as OPG and periapical radiographs, there are various studies that involve intra-oral scanners as well as quantitative, fluorescence, and other new modalities [2,7,8,32,35,73,74,75].

Although OPG is the most widely utilized extra-oral imaging modality in dental care, more standardized standards should be employed to prevent any error owing to image quality, patient orientation, or magnification. To guarantee the creation of a valid set of data, radiographs collected with various OPG equipment should be assessed collaboratively [76]. The research to date frequently commits the mistake of obtaining data from a single imaging method, which is problematic since distinct models are developed for each machine, and it is possible that a model for one device will not apply to other devices. AI models that were trained with manually cropped radiograph data are likewise another problem, since the algorithms may not interpret images without any specific region of interest [1,76]. 

Even if an OPG with proper acquisition techniques is taken, an ideally taken OPG may have its own limitations and difficulties that affect both the clinician’s decision and the precision of AI models. For instance, some scenarios for these OPGs are as follows [77,78]:

- Individuals with maxillofacial disorders or anatomical variations that are unable to maintain an upward spinal posture;
- Patients with severe Class II or Class III malocclusions (due to the inability to position both jaws within the focal trough at the same time);
- Due to difficulties situating the left and right sides of the face inside the focal trough because of facial skeletal asymmetry, only one side of the face may be clearly seen on the radiograph;
- Patients with moderate or severe periodontitis may find it difficult to bite the groove of the biting block. As mobile teeth have a tendency to tilt/move during biting, a cotton roll might be indicated to be put between the upper and lower incisors. Although this seems to be a solution for their acquisition, artefacts related to increased distance affect image quality;
- Even within the same OPG the image magnification changes, often because of anatomical variations and defects. The horizontal plane exhibits more distortion than the vertical plane, which might affect the interpretation of the OPG;
- The OPG’s diagnostic quality is impacted by the image’s tomographic characteristics. The focal trough measures approximately 20 mm in the lateral regions and 10 mm in the anterior area, and only those structures located inside the focal trough are clearly visible on an OPG. Any structures that will be examined outside of this focal trough might cause underdiagnosis;
- Due to superimpositions, bone loss and carious lesions that are localized at the interproximal areas cannot be demonstrated by OPG images. As those superimpositions are more common and problematic in the premolar region, even tooth segmentations may have lower success;
- Ghost images and double shadows are two of the phenomena of OPG imaging that drastically affect interpretation of radiographs.

The aim of this study is to create a deep learning model that can provide automatic segmentations of teeth, dental caries, dental restoration, crown–bridge restorations, dental implants, root canal fillings, and residual roots with a high Dice similarity coefficient value on OPGs that were acquired from three different OPG units to significantly reduce the time spent by dentists on radiological evaluations.

## 2. Materials and Methods

This study was ethically approved by the Health Sciences Ethics Committee of the Near East University Ethics Review Board (YDÜ/2022/108-1651) in December 2022.

In order to eliminate any biases that might cause a negative effect on the generalizability [79], the whole OPG database of the faculty of dentistry, a total of 8138 OPGs that were acquired by 3 different OPG devices, were obtained from the archive of the Near East University, Department of Dentomaxillofacial Radiology.

The exclusion criteria of OPGs were as follows:

- Presence of motion artefacts;
- Presence of removable dentures;
- Presence of fixed orthodontic appliances;
- Presence of ghost images due to glasses, earrings, piercings, and hearing aids;
- OPGs of edentulous patients;
- OPGs with positioning problems (head tilted downwards/upwards, head twisted to one side, head tipped, etc.).

Of the OPGs, 442 were excluded, and 7696 images that were suitable for the study were included.

Following the obtainment of the images in a DICOM format, they were converted into PNG files and transferred to the segmentation tool’s database, Computer Vision Annotation Tool (CVAT), for the segmentation process. 

All teeth, crown, and bridge restorations, dental implants, composite and amalgam fillings, dental caries, residual roots, and root canal fillings were manually segmented by 2 dentomaxillofacial radiologists with the manual drawing semantic segmentation technique.

**Figure 1:**
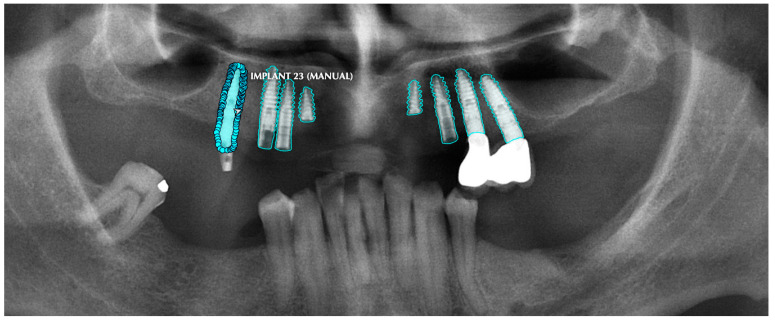
Manual segmentation process of the dental implants. Note the precision for the dental implant’s grove segmentation in order to achieve higher accuracy and DSC.

All of the objects mentioned above were segmented by determining their margins by creating points, and the model was trained separately for each of the structures with those segmentations (Figure 1).

We resized all of the images in the dataset to 512 × 1280 pixels and created our algorithm with the U Net interpretation of the Python computer language. We split all of our segmentations, with 80% in a training set, 10% in a validation set, and 10% in a test set. The most successful model that performed best on the test set was selected. For statistical analysis, the Dice similarity coefficient (DSC) and accuracy values were calculated.

The DSC, an indicator of how identical objects are, was utilized to determine the algorithm’s score. The DSC is calculated by dividing the total area of the two variables by the size of the overlap between the two segmentations. Similar to precision, the DSC measures the number of true positives discovered while additionally penalizing the approach for false positives. The denominator, which includes both the total number of positives and only the positives that the approach identifies, is the sole distinction. As a result, the DSC additionally penalizes for the positives that an algorithm or approach missed [80,81].

## 3. Results

**Figure 2:**
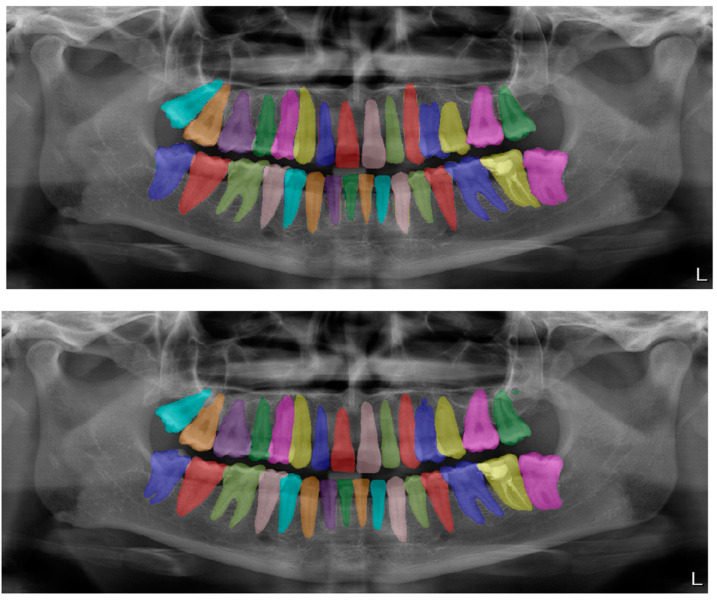
Automatic segmentation of the teeth. Manual segmentation (**upper image**) and automatic segmentation (**lower image**) can be seen above. Each tooth has a unique label according to FDI World Dental Federation notation.

**Figure 3:**
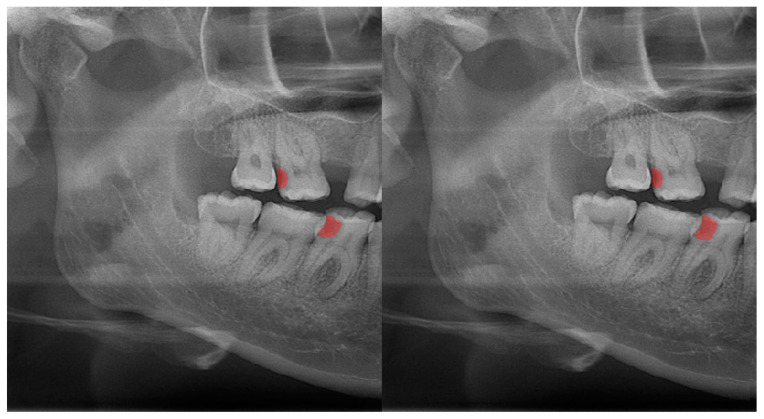
Automatic segmentation of the carious lesions at the upper-left second molar and lower-right first molar. Manual segmentation (**left**) as well as automatic segmentation (**right**) can be seen above.

**Figure 4:**
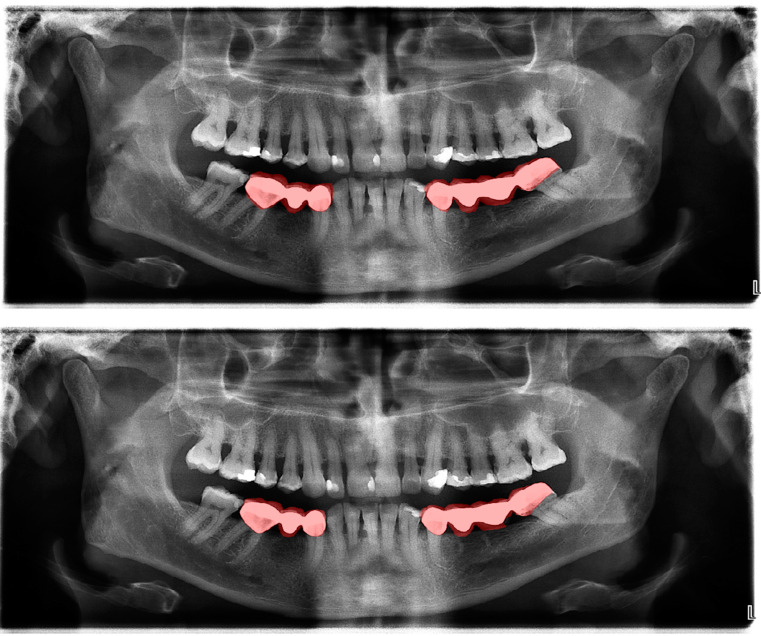
Automatic segmentation of the bridges. Manual segmentation (**upper image**) and automatic segmentation (**lower image**) can be seen above.

**Figure 5:**
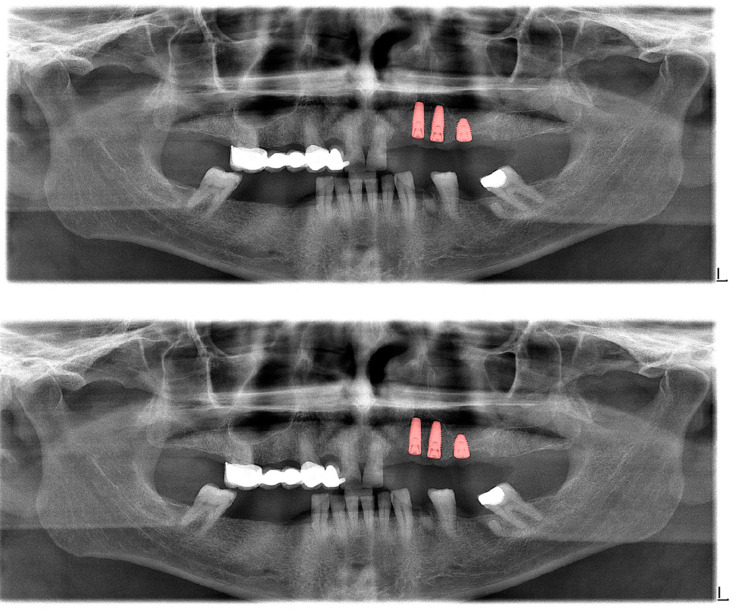
Automatic segmentation of dental implants. Manual segmentation (**upper image**) and automatic segmentation (**lower image**) can be seen above.

**Figure 6:**
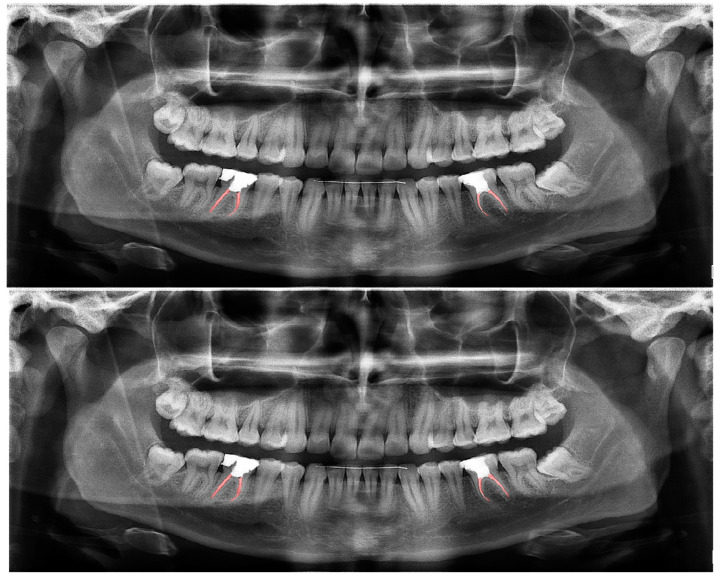
Automatic segmentation of root-canal fillings. Manual segmentation (**upper image**) and automatic segmentation (**lower image**) can be seen above.

**Figure 7:**
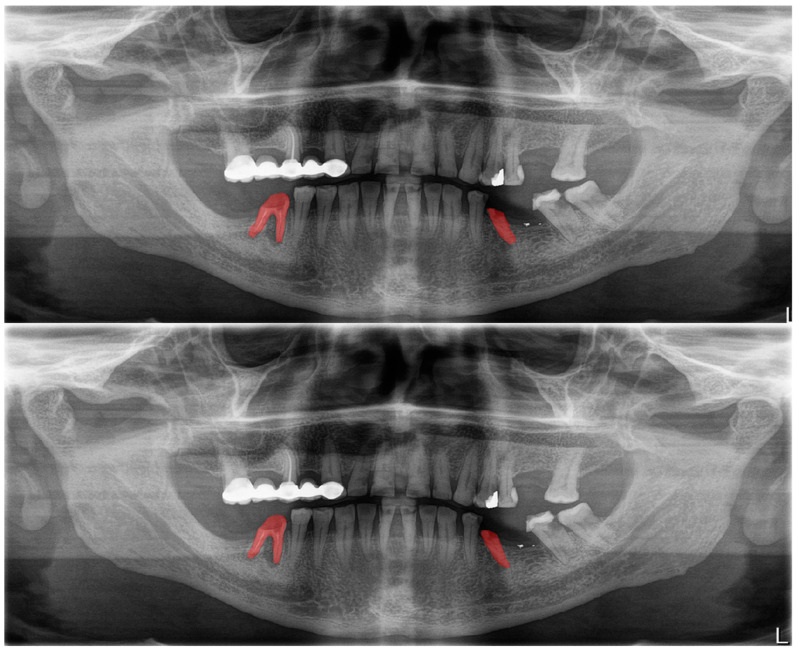
Automatic segmentation of residual roots. Manual segmentation (**upper image**) and automatic segmentation (**lower image**) can be seen above.

**Figure 8:**
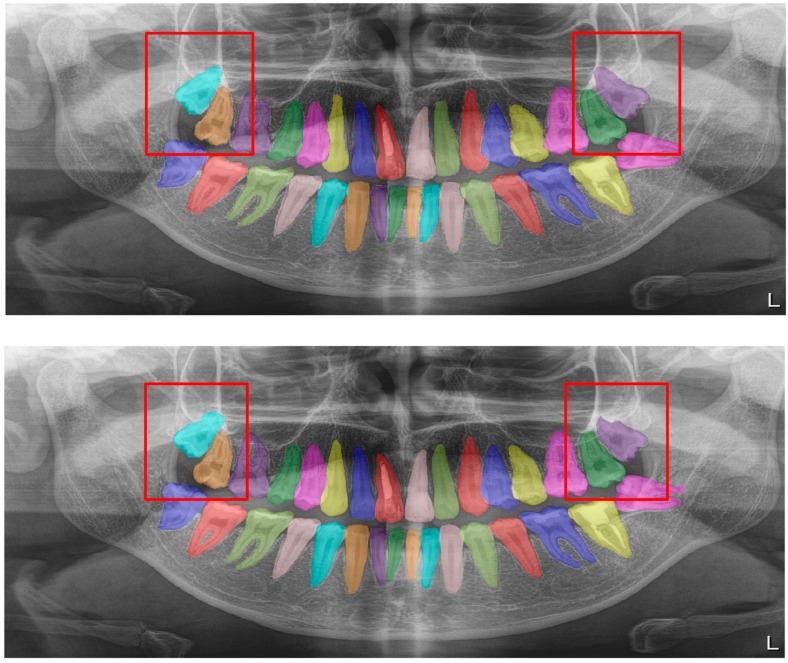
Erroneous automatic segmentation at the maxillary left third molar due to the superimposition between the maxillary sinus floor and root apices of the tooth. Manual segmentation (**upper image**) and automatic segmentation (**lower image**) can be seen above.

**Figure 9:**
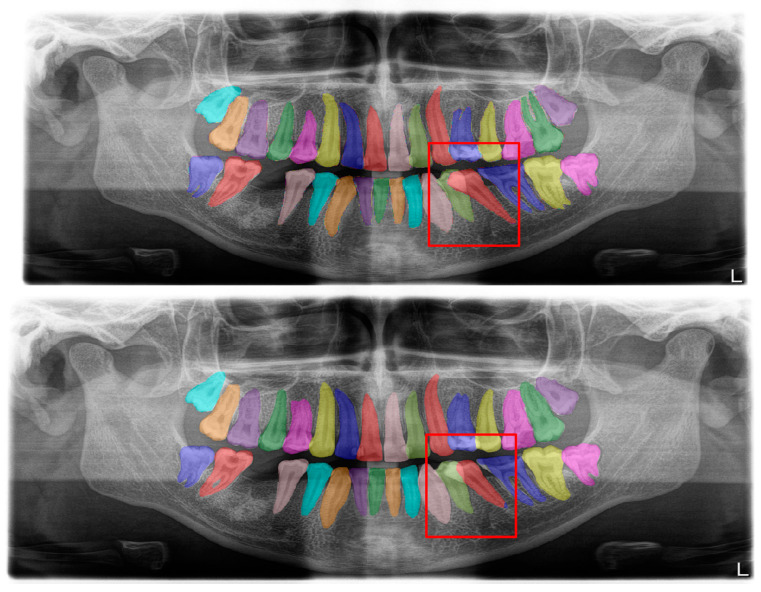
Erroneous automatic segmentation at the mandibular left second molar due to the superimposition between the mandibular left first and mandibular left second premolars. Manual segmentation (**upper image**) and automatic segmentation (**lower image**) can be seen above.

**Figure 10:**
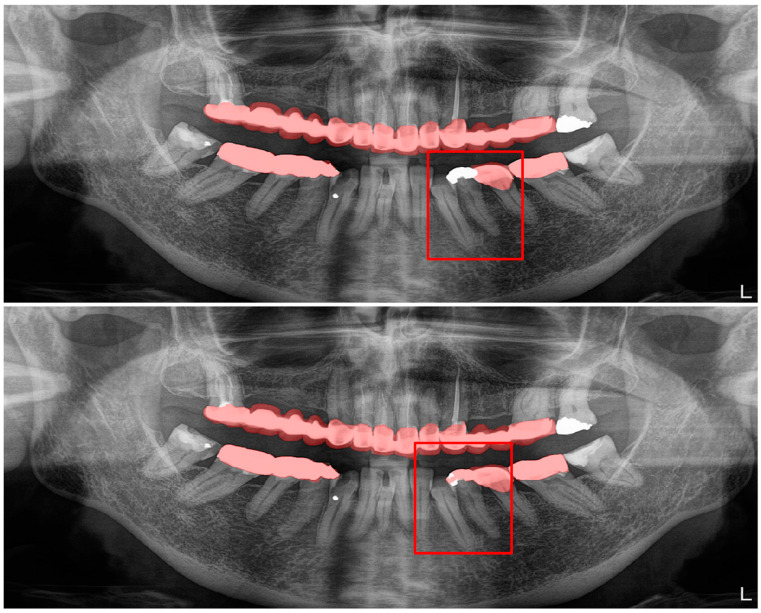
A wide amalgam restoration in the mandibular left first premolar tooth was mis-segmented as a crown restoration. Manual segmentation (**upper image**) and automatic segmentation (**lower image**) can be seen above.

**Figure 11:**
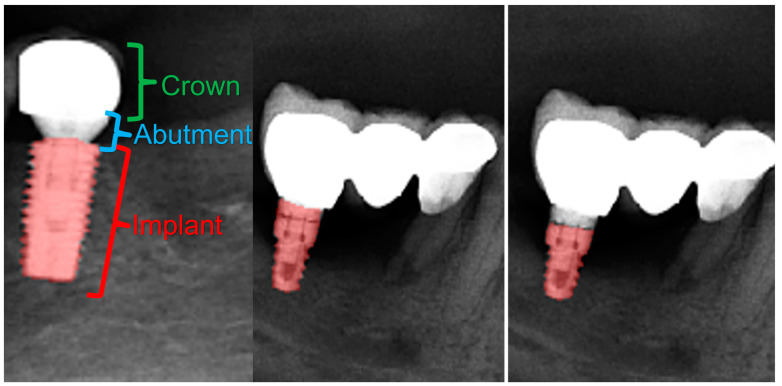
Crown–abutment and implant parts of a dental implant with a crown (**left**). Manual segmentation of the dental implant (**middle**) and automatic segmentation of the dental implant (**right**). Note that the abutment part and the superior portion of the implant were not segmented by our model in this case.

**Figure 12:**
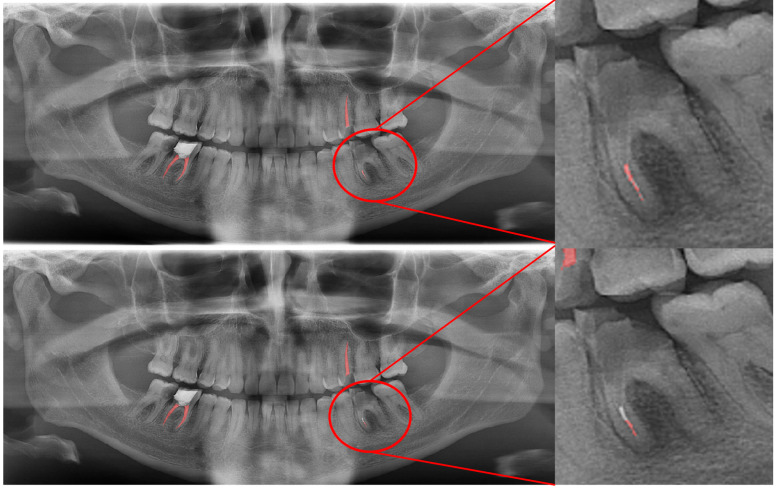
Imperfect segmentation of a gutta-percha is seen at the mandibular left first molar tooth’s mesial root. Manual segmentation (**upper image**) and automatic segmentation (**lower image**) can be seen above.

The calculated DSC values across all OPGs (Table 1) were 0.85 for the teeth, 0.88 for dental caries, 0.87 for dental restorations, 0.93 for crown–bridge restorations, 0.94 for dental implants, 0.78 for root canal fillings, and 0.78 for residual roots. Manual segmentations and successful automatic segmentations of the model are given in Figure 2, Figure 3, Figure 4, Figure 5, Figure 6 and Figure 7, while common erroneous automatic segmentations with the most possible reasons are given in Figure 8, Figure 9, Figure 10, Figure 11 and Figure 12.

Most of the erroneous segmentations occurred due to the limitations of OPG devices, and some of the examples are as follows:

In Figure 8, a more successful automatic segmentation is observed at the maxillary right third molar than the maxillary left third molar, and a missing segmented area in the form of a notch is observed. As most of the upper third molars were superimposed on the floor of the maxillary sinus and zygomatic process of the maxilla, erroneous segmentations were inevitable. In Figure 9, an erroneous automatic segmentation at the mandibular left second molar due to the superimposition between the mandibular left first and mandibular left second premolars can be seen.

In Figure 10, it can be seen that a wide amalgam restoration in the mandibular left first premolar tooth was mis-segmented as a crown restoration. The mis-segmentation of wide amalgam restorations was seen in a total of five OPGs. 

Although the DSC for dental implant segmentation was 0.94 in our study, after checking the output data it was seen that the implant abutments were segmented as dental implants or crowns (Figure 11).

In Figure 12, an erroneous segmentation of an unsuccessful root canal filling can be seen. While our model had a fair DSC value for successful root canal treatments, it was seen that cases with inadequate root canal fillings and gutta-perchas that superimposed with the neighboring teeths’ roots caused a relatively lower DSC value.

## 4. Discussion

In this study, semantic segmentation was performed on OPGs. Semantic segmentation is the classification of all of the different structures on an OPG, namely teeth, implants, fillings, caries, root remnants, and canal fillings, by marking each pixel. U-Net architecture was used to reveal this. U-Net is a convolutional neural network developed at the Freiburg University Computer Science Department for segmentation in image processing studies in biomedical fields [10,11,12,17,20,21,26,30,31,34,38,50,64,66,67,70,71,82,83,84,85,86].

Although the high DSC and accuracy rates demonstrated the highly convenient nature of automatic segmentation, there are numerous pitfalls that we would like to discuss in order to elaborate both the limitations of OPGs and the automatic segmentation of 2D images [87,88,89,90]. Most of the pitfalls were associated with the geometrical limitations of 2D images, but, in order to be more precise, detailed explanations for the pitfalls were as follows:

In tooth segmentation, it has been observed that the segmentation of the root apices of the maxillary third molars, especially those that are impacted in a vertical position, in cases where the root apices are superimposed with the maxillary sinus floor, is incorrectly automatically segmented at different degrees. This is one of the reasons for erroneous segmentation, which reduced the Dice score in our study, albeit only by a small amount. The superposition, which we have seen especially in the premolar region on OPGs, actually shows a limitation of OPGs, not a deficiency of our model (Figure 9). The primary reason why our model could not achieve a perfect result in tooth segmentation is because it is almost impossible to avoid superimpositions on OPGs, especially in premolar teeth, and also because patients with crowding are included in the study. Several studies excluded patients with orthodontic problems; however, one of our main goals was to evaluate the success of our model in the general population, since there will not be any exclusions in dental clinics [91,92].

In crown–bridge segmentations, several large amalgam fillings have been mi-segmented as crown restorations in some cases due to both their width and metallic opacities. To avoid this type of error, amalgam restorations and composite restorations can be segmented with separate labels, and more OPGs that have both amalgam restorations and crown–bridge restorations can be included into the dataset.

In a systematic review conducted by Revilla-Leon et al., it was reported that the automatic segmentation of dental implants by AI models was between 93.8% and 98% in the literature [93]. Similar to the literature, our model was successful, with a DSC value of 0.94. Due to both their external structures with grooves and metallic opacities, dental implants were not mis-segmented as any anatomical structures or restorations, and the automatic segmentation had an almost perfect DSC. When the reasons for the relatively lower DSC in implant segmentation were examined, it was seen that our model randomly segmented the implant abutment in some OPGs and not in others; therefore, it is fair to state that, in further studies, segmentations of dental implants, abutments, and the crowns on implants via three separate labels might increase the DSC, as mis-segmentation between the abutment and the implant will not be present.

In root canal filling segmentations, the number of erroneous situations were higher than the rest of the segmentations as there were multiple limitations. The pitfalls in automatic segmentation for this study’s dataset of the root canal fillings were as follows: inadequate fillings that were carried out with a single or several gutta-perchas, root canal fillings of multirooted teeth that were superimposed with an adjacent tooth, gutta-perchas that did not extend through the entire root canal, and cases in which the restoration in the pulp chamber was misinterpreted and segmented as gutta-percha; however, despite these three limitations, it was seen that the automatic segmentation of our model had more precise and sharply demarcated segmentations than the radiologists did. In root canal filling segmentations, the only limitation was related to the superimpositions, as the residual roots that were superimposed with the neighboring structures were not automatically segmented by our model.

Although it was possible to achieve a higher DSC, we preferred to test our model’s success in a retrospective dataset so that no abnormalities were excluded. Multiple studies are present in the literature that avoided any controversies that might be caused by superimpositions, and in those studies a higher DSC was achieved. For instance, Bayraktar et al. conducted caries detection on bitewing radiographs, and thanks to the bitewing radiographs they excluded the possibility of any superimpositions as the modality is superior in interproximal caries detection [94]. Moreover, Fontenele et al. conducted a similar study, excluding the cases that have metal/motion artefacts by CBCT for the detection of caries [95]. Zhu et al. conducted tooth segmentation for ectopic eruptions and excluded any cases that had an extraction history, periapical periodontitis, or the presence of cystic lesions;, and they also excluded poor-quality OPGs [66]; however, in our study, we only excluded the metal/motion artefacts that create a challenging image for interpretation, even for radiologists.

Furthermore, all of the studies that were mentioned above and the studies that were conducted by Sheng et al. and Ying et al. used only a single imaging unit, which might cause a bias as the models tend to learn the patterns that are characteristic for each imaging unit [67,96]. In order to eliminate this bias, we conducted our study with three different OPG units that have different imaging parameters. One of the only studies that used several OPG units with different models was Schneider et al.’s study, in which they built 72 models with 6 different deep learning network architectures for 1625 bitewing radiographs [31].

This study has several limitations: First of all, although we conducted our study with three different OPG units, the success of our model does not claim a generalizability for all OPG units that can be found on the market [26,31]. Secondly, we concentrated on the DSC values of the segmentations in order to evaluate the success of our model. There are several more reliable metrics, such as pixel accuracy and intersection-over-union (the Jaccard index); however, we used the DSC as it is not only a measure of how many positives were found but it also penalizes for false positives. Additionally, theory states that the DSC and Jaccard index approximate each other relatively and absolutely [97,98]. Thirdly, although the dataset contained OPGs of participants that have different nationalities and ethnic backgrounds we collected the data from a single center, which probably had a negative effect on the generalizability of the model [76]. Last, but not least, it was assumed that increasing the number of OPGs will significantly increase the accuracy, DSC, and robustness of our model; however, in a study that was conducted by Lei et al. [99], how robustness changes with increased amounts of training data for several representative neural networks on different datasets was investigated. They stated that, with increased amounts of training data, both accuracy and robustness improve initially; however, there exists a turning point after which accuracy keeps increasing while robustness starts to decrease. Thus, it is possible that our assumption of using excessive amounts of OPGs might have actually deteriorated the robustness of our model.

In this study, we would like to emphasize the success of our model in multiple tasks on OPGs while discussing the limitations. It must be remembered that, for any AI application in DMFR, we have to maintain a better standardization for both 2D and 3D imaging (such as patient positioning), a bigger dataset (>1000), public datasets from multiple institutions, higher computational power, unsupervised/semi-supervised learning instead of supervised learning, prospectively collected data instead of retrospectively collected and preprocessed data, and randomized controlled trials.

## 5. Conclusions

Artificial intelligence applications in dentomaxillofacial radiology is a fast-processing branch that has had exceptional success in its early stage. With faster and automated diagnosis on 2D and 3D dental images, dentists will have higher diagnosis rates in a shorter time. Although AI applications are not routine in dental clinics, future clinics will certainly be integrated with most of these implementations. 

## Figures and Tables

**Table 1:** The calculated Dice similarity coefficient values of teeth, dental caries, dental restoration, crown–bridge restorations, dental implants, root canal fillings, and residual roots segmentations.

Structure	DSC
Tooth segmentation (Figure 2)	0.95
Dental caries (Figure 3)	0.88
Dental restoration	0.87
Crown–bridge restorations (Figure 4)	0.93
Dental implants (Figure 5)	0.94
Root canal fillings (Figure 6)	0.78
Residual roots (Figure 7)	0.78

## Data Availability

The datasets used and/or analyzed during the current study are available from the corresponding author on reasonable request. The data are not publicly available due to privacy/ethical restrictions.
